# ASSOCIATION BETWEEN NEIGHBORHOOD PHYSICAL ENVIRONMENT SATISFACTION AND AGING ATTITUDES IN OLDER CHINESE ADULTS

**DOI:** 10.1093/geroni/igae098.3796

**Published:** 2024-12-31

**Authors:** Sophie Zhijing Xu, Dan Zhao, Weiyu Mao, Jing Wang, Zhongfang Yang, Yaguang Zheng, Bei Wu

**Affiliations:** New York University, New York, New York, United States; Shandong University, Jinan, Shandong, China (People’s Republic); University of Nevada, Reno, Reno, Nevada, United States; University of New Hampshire, Durham, New Hampshire, United States; Fudan University, School of Nursing, Fudan University, Shanghai, China (People’s Republic); New York University, New York, New York, United States; New York University, New York, New York, United States

## Abstract

With the aging population and increasing prevalence of chronic diseases in China, understanding the factors influencing aging attitudes among elderly individuals is crucial. This study examines the role of neighborhood environment satisfaction in shaping aging attitudes among elderly Chinese individuals with chronic diseases. Using data from the latest China Longitudinal Aging Social Survey (CLASS, 2020), demographic characteristics including gender, marital status, education level, residence type, employment status, and age were analyzed. Regression analysis explored associations between aging attitudes and satisfaction with various neighborhood environment factors, controlling for demographic variables. The sample included 7,111 participants aged 60 years and older. Among them, 5,574 (78.38%) had chronic diseases. Higher satisfaction with road conditions (β = 0.815, P < 0.001) and accessibility facilities (β = 0.339, P = 0.003) showed significant positive associations with attitudes toward aging in this group. Conversely, none of the neighborhood environment satisfaction factors were significantly associated with aging attitudes among older adults without chronic diseases. This study underscores the importance of neighborhood environment satisfaction in shaping aging attitudes among elderly Chinese individuals with chronic diseases. Creating age-friendly environments and tailored interventions can foster positive aging experiences and well-being in this demographic. These findings have implications for policymakers, urban planners, and healthcare professionals striving to create supportive environments conducive to healthy aging in rapidly aging societies like China.

